# Patients’ willingness to pay for health care quality improvement under universal healthcare coverage in Egypt

**DOI:** 10.1186/s42506-025-00195-x

**Published:** 2025-09-02

**Authors:** Hebatullah H. Rozza, Taghareed A. Elhoseny, Safaa H. Abbas, Rasha A. Mosallam

**Affiliations:** 1 High Institute of Public Health, Alexandria University, Alexandria, Egypt; 2 Alexandria Clinical Research Administration, Alexandria Health Affairs Directorate, Alexandria, Egypt

**Keywords:** Willingness to pay, Quality improvement, Cost sharing, Contingent valuation, Payment card, Universal health coverage

## Abstract

**Background:**

In economics, the word “willingness to pay” refers to the highest amount that an individual would be willing to pay, give up, or exchange to obtain goods or services or to avoid something undesirable. It can be applied in healthcare as a way to evaluate the worth of improving the quality of health services. This study aims to assess patients’ willingness to pay (WTP) for healthcare quality improvement among hospitalized patients in two hospitals in Egypt.

**Methods:**

Four-hundred and twenty-six patients were asked to provide a rating for different quality attributes. Patients were presented with a hypothetical scenario and asked about their WTP for a monthly insurance premium to benefit from improving each quality attribute. WTP was elicited using the payment card (PC) response format.

**Results:**

Fifty-two percent of studied patients were not willing to pay to improve healthcare quality attributes. Fifty percent of those who were willing to pay were certain to pay. The most commonly stated reasons for unwillingness to pay were “being governmental responsibility” and “household cannot afford” (43% and 36.8%, respectively). Patients were willing to pay the highest amount of money to improve the quality attributes “competence,” followed by “outcome” and “doctor-patient relationship” (491.03, 465, and 423 LE, respectively). For all quality attributes, except for waiting time and availability of supplies and equipment, the amount of money the patients were willing to pay increased significantly as the perceived quality of that attribute reduced. Advancing age had a significant negative association with WTP (*p* = 0.002). Also, advancement in education was associated with significantly higher WTP (*p* < 0.001). Those with health expenditures ranging from 2000 to < 6000 LE per month were 3.38 times more willing to pay than those with health expenditures ranging from 200 to 1000 LE (*p* < 0.001).

**Conclusion:**

WTP for quality improvement among study participants was low, being the lowest among the elderly and lower-educated individuals. Community financing should not be a method for funding quality improvements except for a few quality attributes such as doctor-patient communication and increased doctor competence. This should be coupled with clear exemption criteria for those unable to pay.

## Introduction

Since the 1980 s, the majority of low- and middle-income countries faced a reduction in the quantity and quality of publicly financed health services. This resulted in the implementation of user fees, which are charges levied at the point of use to act as a sustainable financing source for improving the quality of services [1]. Proponents of user fees have claimed that the money raised will enhance the quality of services provided, making up for the reduction in consumption that results from increasing prices [1, 2]. They also claimed that patients are usually willing and able to pay for health services [3–5]. The failure of cost-recovery systems’ exemption programs, in particular, made it clear that access to health services would be reduced for a significant portion of the population [1, 2]. For example, using user fees in primary care services resulted in higher utilization of preventive services and lower utilization of high-level costly services [6–8]. Two reviews reported a significant reduction in the utilization of health services upon introducing or increasing user fees [1, 9].

To study the population’s behavior toward introducing user fees, most studies assessed the real patients’ responses to different pricing strategies [4]. Few studies assessed populations’ preferences using contingent valuation methods (CV). Contingent valuation (CV) is a survey-based, hypothetical method to determine monetary valuations of a service where either no market exists or the market’s price information does not reflect the actual costs of the service. To measure the value that patients place on health services quality attributes, patients are asked how much money they would be willing to pay to improve one of those attributes, for example, waiting time.

A hypothetical scenario is presented to the patient, and then the willingness to pay (WTP) question is framed in one of four formats: the open-ended format in which the respondent is asked, for example, “what is the maximum amount of money they would be prepared to pay for reducing the waiting time of the service?”; dichotomous choice in which the respondent is asked whether or not he would pay a specified amount, with possible responses being “yes” or “no”; the bidding game where the respondent is provided a certain amount of money, called the bid, and the bid amount is varied across respondents, and the only information obtained from each individual is whether their maximum WTP is above or below the bid offered to them; and the payment card (PC) technique using a payment scale (PS) in which respondents are presented with a range of bids and asked to circle the amount that represents the most they would be willing to pay [10, 11].

Hospital quality attributes for which patients’ WTP was elicited in previous studies were physical proximity of the hospital, accessibility of providers, competence of providers, cost of services, environment cleanliness, and availability of medical supply and equipment [4, 12–14]. In public hospitals in Bangladesh, a closer physician–patient relationship, increased drug availability, and increased chances of recovery were the three, out of seven, hospital attributes for which the patients were most willing to pay in an open-ended WTP survey [12]. In Ethiopia, provider competence, followed by availability of equipment and supplies, was the two attributes with the highest WTP among hospital outpatient attendees [14]. Two-thirds of heads of households in Saudi Arabia were willing to pay for improving the quality of public health services when surveyed using dichotomous choice followed by an open-ended question [5]. In a systematic review, availability of appointments, waiting time before seeing the doctor, and the outcome of treatment were the three attributes for which the respondents were most willing to pay [14].

Planning the study design, data collection, and analysis of a WTP survey requires thorough knowledge of the factors affecting WTP, other than patient experience with the quality of provided services. In a systematic review analyzing 62 studies, sociodemographic characteristics, perceived threat, perceived benefit, and perceived barriers were recorded as significant WTP determinants. Income was the most frequently studied and the most frequently significant determinant of WTP. Age and education were the next most frequently studied determinants [15].

Egypt enacted a Universal Health Insurance (UHI) law in 2018 to introduce universal health coverage (UHC) by the year 2030 through a phased approach [16]. Setting the prices for health services under UHC is one of the challenges encountered by the Egyptian government [17]. Pricing health services is a challenging task as it entails a trade-off between sustainability and accessibility [18]. The current study aims to assess patients’ WTP for healthcare quality improvement in a health insurance hospital and a private hospital in Alexandria. This will provide a demand-based approach to describe consumer preferences by studying their potential purchasing behavior. This will allow for improving the services based on consumers’ preferences and will allow for realistic pricing of services.

## Methods

### Study design

A descriptive cross-sectional study design was employed.

### Case selection

Assuming that 50% of patients are willing to pay for quality improvement, with a confidence level of 95% and a degree of precision of 5%, the minimum required sample size is 384. Stratified random sampling was used to recruit patients. Stratification was performed by the hospital department. Each stratum was determined by a proportional allocation method according to the number of hospital departments’ discharges in the previous year. In each department, patients were selected using a simple random sampling technique using a random number table, excluding patients who were seriously ill or refused to participate. A total of 426 adult inpatients (206 from health insurance hospital and 220 from the private hospital) who were cognitively able and willing to participate in the survey were included.

### Data collection tools

Data was collected through a face-to-face interview using a predesigned questionnaire based on reviewing previous relevant literature [5, 11, 12, 14, 19–23]. Data was collected from January to March 2024.

The questionnaire is composed of three sections. The first is patient sociodemographic data such as age, sex, household income, education, employment, and perceived own health status. The second section was the hospital quality attributes that can influence the patient WTP and their level. Examples of quality attributes are as follows: healthcare provider competence, availability of medical equipment and supplies, distance from the hospital, nurse communication, doctor-patient relationship, cleanliness and quietness of the hospital environment, and waiting time. Patients were asked to provide a rating for each attribute based on the assessment of their current visit to the hospital.

The third section is the patient WTP for improvement of each of the hospital quality attributes. WTP was elicited using the PC response format, which offers a range of values to respondents and asks them to choose the maximum amount they would be willing to pay. WTP questions were asked in two stages. First, patients were presented with a hypothetical scenario and were asked about their WTP for a monthly insurance premium in order to benefit from better quality of healthcare services. Then, if they agreed to pay, the interviewer proceeded to the WTP elicitation technique. If they were not willing to pay, they were asked a list of possible reasons.

The starting point of the PS was initially set at the cost-sharing ceiling (300 Egyptian pounds) under the Egyptian Universal Health Insurance law. The end point was derived from the average cost of an inpatient stay in a private hospital in Alexandria Governorate, Egypt. The start and end points of the PS were validated based on the responses of an open-ended pilot study on a sample of patients and the results of a face validity evaluation of 10 experts in healthcare management. After the pilot study, the start point was reduced to 100 Egyptian pounds. Monetary values were displayed in Egyptian pounds.

Improvements in the quality attributes were separately assessed using a decomposed valuation scenario. For example, the patient would be asked, “What is the maximum amount of money that you would be willing to pay to benefit from a hospital similar to this one and located very close to your home?” to assess WTP for the attribute distance to the hospital.

The decomposed valuation method assumes that utility variations following improvements in one attribute do not depend on the levels of other quality attributes. For each respondent, the minimum and the maximum WTP were elicited as follows: the respondent was asked to tick the highest amount he would definitely pay to improve the selected quality attribute (minimum WTP), for example, 100 EGP. Then, he was asked to tick the first amount he would definitely not pay to improve the quality attribute, for example, 800 EGP (maximum WTP).

A follow-up open-ended (OE) WTP question that was bounded by the minimum and maximum WTP indicated by the patient on the PS followed. For example, “You have indicated that you would definitely pay 100 EGP and definitely not pay 800 EGP to improve the selected quality attribute, please write in the amount (between 100 EGP and 800 EGP) that most closely approximates the maximum you would be willing to pay per month to improve the selected quality attribute.”

Certainty surrounding the post-estimation response of the OE WTP question was assessed. Respondents were asked how certain they were about actually paying OE-WTP if asked right now, with response options as follows: (1) totally certain I would pay; (2) pretty certain I would pay; (3) maybe yes, maybe no; (4) probably would not pay; and (5) surely would not pay. Previous research documented an association between actual consumption behavior and higher levels of response certainty [11].

#### Payment scale

**Table:**
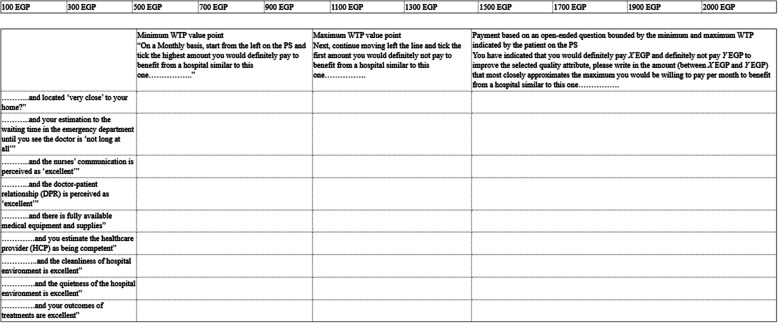

### Data analysis

The raw data was coded and entered into the computer using the SPSS (Statistical Packages for Social Sciences) version 26.0 for Windows. The chi-squared test (*χ*^2^) was used as a test of significance. Whenever the chi-squared test was not valid (more than 20% of expected values have a count less than 5), Fisher’s exact test was used instead. The *t*-test was used as a test of significance for comparison of means. The 5% level was used as a cut-off point value of statistical significance. The binary logistic regression model was used to examine the effect of selected patient characteristics on the likelihood of willingness to pay. The dependent variable was the WTP, and it was coded as (0, 1). The independent variables were age group, marital status, the highest level of education, health expenditure, distance, waiting time, nurse communication, doctor-patient relationship, the competence of doctors, cleanliness, quietness of the hospital, and overall satisfaction. All independent variables were included as categorical variables in the model. The first category of each variable was considered as the reference category.

## Results

About two-fifths of the studied patients were in the age category 35– < 52 years old (39.7%), and most were females (52.6%), married (72.5%), held a university or a postgraduate degree (50%), and rated their health as good (50.0%). Around one-quarter were employed in the private sector, and the other quarter was retired (26.8% and 25.4%, respectively). As for the household head income, around half of the study population (47.8%) had income ranging from 4000 to < 6000 EGP with a mean of 5134 ± 1584.8. Similarly, the highest proportion of household expenditure ranged from 4000 to < 6000 EGP (51.7%) with a mean of 5105 ± 1441.3 EGP. Around two-thirds of the sampled population spent 200 ≤ 1000 EGP on healthcare per month (69.7%), with a mean of 1168 ± 796.7 EGP.

About half of the respondents were not willing to pay for the improvement of healthcare services (52.3%) (Table 1).

**Table 1:** Comparison of patients’ willingness to pay to improve healthcare quality services in a health insurance and a private hospital, Egypt (2024)

**Willingness to pay**	**Health insurance hospital** ***n* = 206** ***n* (%)**	**Private hospital** ***n* = 220** ***n* (%)**	**Total** ***n* = 426** ***n* (%)**	***χ***^**2**^	**P**
Yes	100 (48.5)	103 (46.8)	203 (47.7)	0.127	0.722
No	106 (51.5)	117(53.2)	223(52.3)		

*χ*^2^, chi-square test

**Fig. 1:**
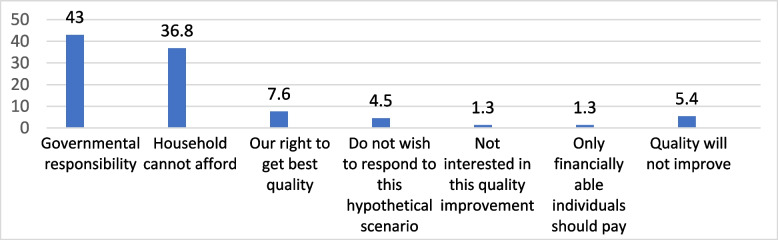
Reasons for unwillingness to pay

The most frequently stated reasons by respondents for unwillingness to pay for quality improvement were “governmental responsibility,” “household cannot afford,” and “Our right to get the best quality” (43.0%, 36.8%, and 7.6%, respectively) (Fig. 1).

**Fig. 2:**
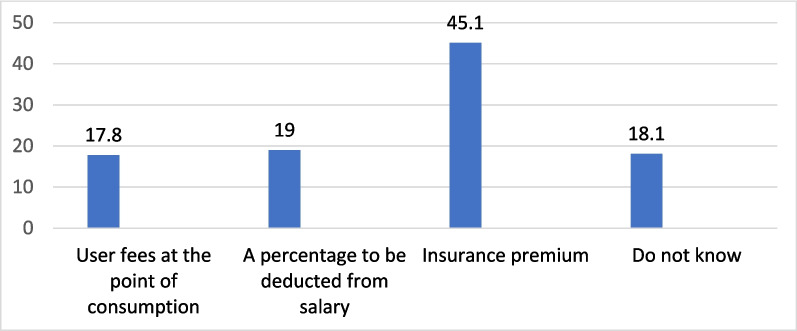
Methods of contribution to finance health services

As for the method of contribution to healthcare finance, about half of the respondents stated that the contribution should be through an insurance premium, followed by a percentage to be deducted from salary (45.1% and 19.0, respectively). The least selected option was user fees at the point of consumption (17.8%) (Fig. 2).

Patients were willing to pay the highest amount of money to improve the quality of the attributes “competence,” followed by “outcome” and “doctor-patient relationship” (491.03 ± 281.1, 465.6 ± 303.1, and 423.03 ± 318.9, respectively) (Table 2).

**Table 2:** Amount of money the study population was willing to pay for the improvement of each quality attribute

**Quality attribute**	**Total** **(mean ± SD)**
Distance	265.2 ± 251.5
Waiting time	291.4 ± 270.1
Nurse communication	331 ± 91.5
Doctor-patient relationship	423.03 ± 318.9
Availability of equipment and supplies	391.1 ± 302.3
Competence	491.03 ± 281.1
Cleanliness	334.2 ± 316.9
Quietness	355.4 ± 690.4
Outcome	465.6 ± 303.1

The age category with the highest percentage of WTP was the category 20– < 35, while that with the lowest percentage of WTP was the category 76– < 83. Concerning marital status, three-fifths of single respondents were willing to pay compared to around half for married respondents and only one-third for divorced individuals and widows (61.3%, 51.5%, and 29.1%, respectively). As for education, as the respondent advances in the level of education, the WTP increases. Only one-third of respondents who were uneducated, with elementary or intermediate education, were willing to pay compared to three-fifths of those with university and postgraduate degrees (31.7% and 58.7%, respectively). Those working in the governmental sector had the highest percentage of WTP (59.5%). As for household head income and household expenditure, all income categories had almost the same percentage of WTP (around half). Concerning health expenditure, those with the highest expenditure category per month “2000– < 6000” had the highest percentage of WTP (80.6% compared to 45.4% for the health expenditure category “200 ≤ 100.” Differences in willingness based on age, marital status, level of education, and categories of health expenditure were statistically significant (Table 3).

**Table 3:** Patient characteristics and willingness to pay in a health insurance and a private hospital, Egypt (2024)

**Sociodemographic** **characteristics**	**Willingness to pay**		**Total** ***n* = 426** ***n* (%)**	***χ***^**2**^
	**Yes** ***n* = 203** ***n* (%)**	**No** ***n* = 223** ***n* (%)**		
Age				14.74*
20– < 35	35 (70.0)	15 (30.0)	50 (11.7)	
35– < 51	81 (47.9)	88 (52.1)	169 (39.7)	
51– < 67	72 (44.7)	89 (55.3)	161 (37.8)	
67– < 83	15 (32.6)	31 (67.4)	46 (10.8)	
Sex				1.26
Female	113 (50.2)	112 (49.8)	225 (52.8)	
Male	90 (44.8)	111 (55.2)	201 (47.2)	
Marital status				16.00*
Single	19 (61.3)	12 (38.7)	31 (7.3)	
Married	159 (51.5)	150 (48.5)	309 (72.5)	
Divorced, widow	25 (29.1)	61 (70.9)	86 (20.2)	
Highest level of education				23.77*
Not educated, elementary, intermediate	13 (31.7)	28 (68.3)	41 (9.6)	
High school	28 (45.9)	33 (54.1)	61 (14.3)	
Two years institute	37 (33.3)	74 (66.7)	111 (26.1)	
University degree, postgraduate degree	125 (58.7)	88 (41.3)	213 (50.0)	
Rate health				2.91
Excellent, very good	59 (48.0)	64 (52.0)	123 (28.9)	
Good	108 (50.7)	105 (49.3)	213 (50.0)	
Fair, poor	36 (40.0)	54 (60.0)	90 (21.1)	
Employment				8.66
Unemployed	43 (42.2)	59 (57.8)	102 (23.9)	
Self-employed	9 (39.1)	14 (60.9)	23 (5.4)	
Governmental	47 (59.5)	32 (40.5)	79 (18.5)	
Private	59 (51.8)	55 (48.2)	114 (26.8)	
Retired	45 (41.7)	63 (58.3)	108 (25.4)	
Household head income	*N#* = 172 *n* (%)	*N#* = 175 *n* (%)	*N#* = 347 *n* (%)	0.511
2000– < 4000	61 (51.7)	57 (48.3)	118 (34)	
4000– < 6000	79 (47.6)	87 (52.4)	166 (47.8)	
6000– < 10,000	32 (50.8)	31 (49.2)	63 (18.2)	
Health expenditure	*N#* = 190 *n* (%)	*N#* = 183 *n* (%)	*N#* = 373 *n* (%)	14.61*
200– < 1000	118 (45.4)	142 (54.6)	260 (69.7)	
1000– < 2000	45 (54.9)	37 (45.1)	82 (22)	
2000– < 6000	25 (80.6)	6 (19.4)	31 (8.3)	

^*^p < 0.05

*χ*^2^, chi-square test; *n#*, number of respondents

Those who rated the cleanliness and quietness of the hospital as being “very bad” were willing to pay about three-folds the amount of those rating the same attributes as excellent. For the attributes “distance,” “doctor’s competence,” “cleanliness,” and “quietness,” it was noticed that WTP amount increases as the rating for those attributes worsens. This increase is statistically significant. Respondents who rated the hospital as being “very far” were willing to pay a mean of 607.14 ± 340.73 EGP to improve that attribute compared to 211.76 ± 207.09 EGP for those who rated the hospital as being “very close.” Respondents who rated the doctor as being not competent were willing to pay double the amount of money compared to those who rated the doctor as being competent (Table 4).

**Table 4:** Mean willingness to pay for different quality attributes’ ratings

Healthcare quality attribute	Mean willingness to pay to improve each attribute ± SD	Significance@
Distance		0.000*
Very far	607.14 ± 340.73	
Far	292.75 ± 229.71	
Close	227.98 ± 225.71	
Very close	211.76 ± 207.09	
Waiting time		0.016*
Very long	205.00 ± 158.9	
Long	221.42 ± 202.3	
Not long	346.25 ± 306.06	
Nurse communication		0.089
Very bad	600 ± 0.00	
Bad	600 ± 442.71	
Good	310.29 ± 279.08	
Excellent	331.00 ± 291.57	
Doctor-patient relationship		0.067
Very bad	1000.00 ± 0.00	
Bad	200.00 ± 70.7	
Very good	400.33 ± 314.01	
Excellent	466.42 ± 324.34	
Competence		0.014*
Not competent at all	1000.00 ± 0.00	
Not competent	1000.00 ± 0.00	
Moderately competent	466.02 ± 285.18	
Competent	494.73 ± 270.06	
Availability of equipment		0.535
Not available	425.75 ± 369.99	
Partially available	562.5 ± 468.8	
Fully available	418.26 ± 300.49	
Availability of supplies		0.000*
Not available	275.92 ± 228.766	
Partially available	541.54 ± 343.41	
Fully available	401.42 ± 251.31	
Cleanliness of hospital		0.015*
Very bad	1000.00 ± 0.00	
Bad	800.00 ± 0.00	
Good	365.62 ± 339.18	
Excellent	276.25 ± 265.76	
Quietness of hospital		0.033*
Very bad	1000.00 ± 0.00	
Bad	657.21 ± 1638.95	
Good	284.29 ± 287.52	
Excellent	322.20 ± 277.21	

^*^p < 0.05

@Significance of *t*-test

About four-fifths of the study population in the private hospital were totally certain to pay (78.6%) compared to only one-fifth in the health insurance hospital. Around two-fifths (40%) of the study population in the health insurance hospital stated that they “surely would not pay” compared to only 1% in the private hospital (Table 5).

**Table 5:** Certainty of willingness to pay reported by respondents in a health insurance and a private hospital, Egypt (2024)

**Certainty of willingness to pay**	**Health insurance hospital** ***n* = 100** ***n* (%)**	**Private hospital** ***n* = 103** ***n* (%)**	**Total** ***n* = 203** ***n* (%)**	***χ***^**2**^
Totally certain to pay	21 (21.0)	81 (78.6)	102 (50.2)	77.3*
Pretty certain to pay	19 (19.0)	14 (13.6)	33 (16.3)	
Probably would not pay	20 (20.0)	7 (6.8)	27 (13.3)	
Surely would not pay	40 (40.0)	1 (1.0)	41 (20.2)	

^*^p < 0.05

*χ*^2^, chi-square test

In the binary logistic regression model, advancement of age was associated with a reduction in the odds of willingness to pay. Being in the age group “67– < 83” was significantly associated with a 78.0% reduction in WTP compared to the age group “20– < 35” which could range from 24.0% up to 94.0%. Being “married” or “divorced or widow” significantly lowers the WTP by 73.0% (ranging from 17.0% up to 91.0%) and 90.0% (ranging from 62.0% to 98.0%), respectively, compared to being “single.” The farther the distance to the hospital, the higher the WTP to improve the quality attributes. Patients who rated the distance to the hospital as being “far” had 3.11 odds of WTP compared to those who considered the distance to be “very close.” Similarly, patients who considered the distance to be “very far” had 3.72 odds of WTP compared to those who considered the distance to be “very close.” Rating the nurse’s communication as “good” was associated with a 60.0% reduction in the WTP compared to rating the communication as excellent (ranging from 25.0% to 78.0%). On the contrary, the lower the score for the relation with the doctor, the higher the odds of WTP. Patients rating the relationship as good had almost twice the odds of WTP compared to those rating this relationship as “excellent.” As for doctor competency, rating the doctor as being “moderately competent” and “not competent/not competent at all” was associated respectively with a 69.0% and 97.0% reduction in WTP compared to rating the physician as competent (Table 6).

**Table 6:** Binary logistic regression of selected studied patients’ characteristics and healthcare quality attribute ratings in relation to willingness to pay

Variable	Odds ratio	95% confidence interval
Age group		
20– < 35®		
35– < 51	0.53	0.207–1.36
51– < 67	0.54	0.203–1.42
67– < 83	0.22*	0.06–0.76
Marital status		
Single®		
Married	0.27*	0.09–0.83
Divorced, widow	0.10**	0.02–0.38
Highest level of education		
Not educated, elementary, intermediate®		
High school	1.00	0.33–3.02
Two years institute	0.73	0.27–1.97
University degree, postgraduate degree	1.52	0.59–3.89
Health expenditure		
200– < 1000®		
1000– < 2000	1.41	0.75–2.64
2000– < 6000	2.26	0.76–6.71
Distance		
Very close®		
Close	1.69	0.85–3.33
Far	3.11*	1.21–7.99
Very far	3.72	0.94–14.72
Waiting time		
Not long®		
Long	0.53	0.27–1.03
Very long	0.39	0.13–1.21
Nurse communication		
Excellent®		
Good	0.40**	0.22–0.75
Bad, very bad	0.45	0.13–1.49
Doctor-patient relationship		
Excellent®		
Good	1.95*	1.01–3.74
Bad, very bad	1.63	0.27–9.65
Competence		
Competent®		
Moderately competent	0.31**	0.18–0.56
Not competent, not competent at all	0.03*	0.00–0.45
Hospital		
Health insurance hospital®		
Private hospital	0.68	0.32–1.42

Model, *χ*^2^ = 96.05; *p* = 0.000. Percent correctly classified = 71.2%. R square = 33.1%. ®Reference category. *p < 0.05. **p < 0.005

## Discussion

User fees as a mechanism of cost recovery in developing countries were advocated by the World Bank (WB) in 1985. Since then, user fees were implemented in many low- and middle-income countries with the support of the World Bank and the World Health Organization (WHO) [24]. Supporters of user fees hypothesize that those fees are a sustainable source of funding health services, especially in low-resource countries. In addition, those fees help improve the quality of provided services and reduce the inappropriate use of those services [1].

The current study aims to assess patients’ WTP for healthcare quality improvement in two hospitals in Egypt. The results revealed that almost half of the patients were not willing to pay for an improvement in the quality of health services. When asked about the certainty of willingness to pay, around half of those willing to pay reported that they were not certain of their willingness. For those who were not willing to pay, around half attributed this to healthcare being the government’s responsibility, and around one-third were unable to afford to pay for quality improvement. The quality attributes for which patients were willing to pay the highest amount were the doctor’s competence, outcomes, and doctor-patient relationship. For all quality attributes, with the exception of waiting time and availability of supplies and equipment, the amount of money the patients were willing to pay increased significantly as the perceived quality of that attribute reduced.

### WTP and its policy implications

The WTP in the current study was lower than that reported in other studies. In 2001 in Sudan, a household WTP survey revealed that 80% of those paying a charge for the health services and 75% for those receiving it free of charge were willing to pay for quality improvements of health services [3]. In Palestine in the period from 2001 to 2003, WTP to improve the quality of health services in primary care units dropped from 78.0% in 2001 to 61.0% in 2003 [25]. In another study conducted among attendants of primary healthcare centers in Palestine, 93.4% declared they were willing to pay higher user fees to benefit from better quality [4]. In a national survey conducted in the Central African Republic, WTP ranged from 64.0% to 81.0% for seven quality attributes for improving child care [26]. In a household survey conducted in 2000, the majority of Bulgarians were in favor of paying for public services with good quality and quick access [27].

The low results of WTP in the current study can have several explanations. One of the explanations is that the current study utilized a PS in eliciting the WTP compared to the closed-ended format used in the Central African Republic and Sudan studies and the dichotomous choice in the Palestinian study [3, 25, 26]. Closed-ended WTP format resulted in significantly higher valuations than the PS formats in a study eliciting the WTP for alternative methods of screening for colorectal cancer [29]. Similarly, the dichotomous choice format has been shown to consistently generate larger values than the PS [28, 29].

Another explanation for the low percentage of respondents willing to pay in the current study is the high level of inflation and the tightening financial conditions in Egypt since the devaluation of the Egyptian pound in March 2022. This resulted in an increase in poverty rates [30, 31]. The GDP per capita in Egypt is US $3457 which places Egypt in the 92nd rank compared to other countries. In their study, Mataria et al. demonstrated that WTP for the attributes geographical proximity and waiting time were most affected by the substantial reduction in patients’ income. This can be explained by the fact that quality attributes perceived to be nonessential luxuries are significantly affected by income reduction. On the other hand, the WTP for essential quality attributes such as drug availability and doctor-patient relationship were not affected by impoverishment [25]. Similarly, in the current study, patients were willing to pay the highest amount of money to improve the quality of the attributes “competence,” followed by “outcome” and “doctor-patient relationship.”

In the current study, household head income was not significantly associated with WTP as opposed to health expenditure. This was explained by Russel as the difference between WTP and ability to pay (ATP) [33]. He proposed that households may cope with the user fees for healthcare by sacrificing other basic needs such as food and education or by borrowing and selling assets. According to the Egyptian Health Insurance law, user fees upon receiving service will be applied but with an exemption for those unable to pay [34]. However, there is no explicit mechanism for defining those who are unable to pay [19]. Thus, national “inability to pay” income criteria should be clearly defined against which people will be judged for exemption. In addition, further research on the mechanisms the patients use to cope with healthcare costs should be conducted.

A third explanation for the low level of WTP is the level of trust in the government in Egypt. According to a report by the Arab Barometer in 2019, 66% of Egyptians reported having a great deal or quite a lot of trust in the government [35]. In another report by the European Bank for Reconstruction and Development, corruption was still perceived as a problem in public and private organizations [36]. In research analyzing data from the fifth wave of the Arab Barometer survey to identify the main determinants of public trust in the government of Egypt, perceiving national institutions as corrupt had a negative impact on the likelihood of trusting the government [37]. In a survey covering 29,526 respondents from 29 countries, an increase in social trust was associated with a greater WTP for more taxes to improve public healthcare [38].

In the current study, around half of the respondents believed that improving the quality of healthcare is the responsibility of the government and not the citizens. Thus, building social trust in the government as well as the health system is a cornerstone for the public to share in funding the healthcare system. According to Gilson, trust in the healthcare system can be built through fairness in the distribution of health resources by ensuring geographical, financial, and cultural accessibility for all and through setting equality goals [39].

In the current study, the percentage of those willing to pay decreased significantly as age advanced and the level of education decreased. Similar findings were reported by Mataria et al. among patients attending primary care units in Palestine and by Al-Hanawi et al. among heads of households in Saudi Arabia [4, 5]. In a systematic review of determinants of WTP for health services, age was a significant determinant for WTP in 30 out of 50 studies, whereas education was a significant predictor in 30 out of 45 studies (60.0% and 66.7%, respectively) [15]. In another review assessing the WTP for national health insurance services in Asia and Africa, the age of the household head was significantly and negatively associated with WTP in 7 out of 19 studies, and education was significantly and positively associated with WTP in 5 out of 19 studies [40].

One of the explanations for this finding is that as people age, their income decreases, resulting in a reduction in their capacity to pay for health services [41]. Although this study did not attempt to measure demand elasticity, we could expect that the demand is elastic among the older population and those with lower education, which could result in a reduction in accessibility among those groups. If policymakers are aiming at equitable accessibility among different population groups, a price discrimination approach should be adopted if user fees are used to co-fund the quality improvements in health services [4, 42]. According to the results of our study, lower user fees should be charged to the elderly and those with lower education attainment. In addition, the government should subsidize the care for price-sensitive groups of the population and closely monitor how the level of demand was affected by the introduction of user fees [43].

The mean value the patients were willing to pay increased significantly as the patients’ ratings for the attributes distance from the hospital, physicians’ competence, hospital cleanliness, and quietness decreased. For example, patients who rated the hospital as “very far” were willing to pay three times as much as those who rated the hospital as “very close.” Similarly, in the logistic regression model, those who rated the hospital as “very far” had 3.7 higher odds of WTP for receiving care compared to those who rated the hospital as “very close.” This finding was similar to those reported in Saudi Arabia and Bangladesh where WTP and the amount of money the patients were willing to pay were inversely related to the rating of different quality attributes [5, 12]. This would be a validation of the WTP methodology used as patients were willing to pay to improve attributes with low-perceived quality. In the current study, we assumed independence between different quality attributes. That is, improvement in one attribute is independent of the improvements over others. Further research should verify the existence of this independence.

### Validity of the results of the contingent valuation methodology

The validity of the contingent valuation methodology has been widely questioned. The contingent valuation methodology in our study has been conducted during a time of economic recession in Egypt. Under tough economic conditions, people may modify their preferences to accommodate only services they consider “essential.” Thus, under those conditions, unwillingness to pay for quality improvement of certain service attributes should not be directly interpreted as a lack of preference for those attributes [4]. Economic and social conditions should be considered when interpreting the results of WTP surveys.

Our study attempted to improve the validity of the WTP survey through several techniques. First, to reduce the “hypothetical bias” which is the extent to which the WTP scenario and the valuation task are believable by the respondents [22], the valuation scenario in the current study was insurance based (i.e., insurance premium) rather than a user-based scenario (i.e., user fees at the point of consumption). This approach made the scenario more realistic as the Universal Health Insurance Law was issued in 2018. Until the time of the study, it was piloted in several governorates [16]. The Egyptian Healthcare Authority is planning a phased implementation of the new universal health insurance system until universal health coverage encompasses all governorates by 2030. Thus, the insurance-based scenario was realistic and understandable for the respondents [44].

To reduce the respondents’ cognitive load and improve the accuracy of the results, the decomposed valuation scenario approach was employed instead of the holistic approach in which the respondents are asked for the monetary value for quality improvement in general [4]. O’Brien et al. recommended that if multiple attributes are to be valued, three or four most valuable ones should be identified. WTP exercise should be based on each attribute separately [5, 45].

The current study utilized the PS methodology for eliciting WTP values. Compared to other CV methods, the PS methodology avoids starting point bias inherent in the bidding game and dichotomous choice formats since the questions do not offer an initial bid. Also, it avoids the “yea-saying bias” in dichotomous choice surveys. PS also reduces the cognitive load on the respondents since they are offered a predefined set of values. On the other hand, PS entails a range bias, where the range of bids affects the valuation. Respondents may choose to report a comparatively higher WTP in a hypothetical exercise if their true WTP value is relatively low compared to the values displayed on the PS and vice versa [11, 46].

Respondents may be able to elicit values that are closer to their “true” WTP values if the range is more realistic. In our study, the range of values was piloted among several patients to ensure that it is realistic. In addition to selecting a valuation from the PS, respondents were asked to choose from the PS the amount they were definitely willing to pay (minimum WTP) and the amount they were definitely not willing to pay (maximum WTP) for each attribute. This was followed by an open-ended question to select a value between the minimum and the maximum WTP to further reduce the range bias.

To predict the actual consumption behavior, a post-estimation response certainty question was asked. Respondents were asked how certain they were about paying for the improvement of different quality attributes if they were asked right now. Among those willing to pay in our study, only half of the respondents were completely certain they were willing to pay. Four-fifths of those willing to pay were private hospital respondents, and one-fifth were insurance hospital respondents. This could be an indication of the respondent’s actual behavior in real situations where the WTP percentage would be much lower than that reported, and we can even expect the monetary values to be lower [34].

### Limitations of the study

Despite being the first study to measure WTP among the Egyptian population, the current study has selected an “in-site” sample rather than a household survey. Thus, those who did not attend the healthcare facilities were not represented. Moreover, not all facilities were represented as, for example, patients attending the Ministry of Health and University healthcare facilities were not included. Those groups are usually socially and economically disadvantaged compared to patients currently covered by the social health insurance system or attending private facilities and paying out of pocket. Further research should be conducted covering wider segments of the population and using household surveys.

## Conclusion

The WTP for quality improvement of the survey participants was lowest among the elderly and those with lower education. Thus, if user fees were utilized to co-fund the quality enhancements in health services, a price discrimination strategy has to be implemented to prevent a reduction in the accessibility of services for price-sensitive groups such as the elderly and those with lower education. The quality attributes that people were most willing to pay for were the doctor-patient relationship, outcome, and competence. As the perceived quality of the attribute declined, the patients’ WTP rose significantly.

Based on the current study, several implications can be drawn out. Firstly, policymakers should not depend on community financing to fund quality improvements except for a few quality attributes, such as doctor-patient communication and increased doctor competence. This should be coupled with monitoring accessibility to health services. Additionally, a price discrimination approach should be adopted if user fees are used to co-fund the quality improvements in health services. National “inability to pay” income criteria should be clearly defined against which people will be judged for exemption. Moreover, further research on the mechanisms patients use to cope with healthcare costs should be conducted.

## Data Availability

No datasets were generated or analysed during the current study.
